# Neurofeedback to enhance sleep quality and insomnia: a systematic review and meta-analysis of randomized clinical trials

**DOI:** 10.3389/fnins.2024.1450163

**Published:** 2024-11-06

**Authors:** Jose I. Recio-Rodriguez, Mei Fernandez-Crespo, Natalia Sanchez-Aguadero, Jesús Gonzalez-Sanchez, Irene A. Garcia-Yu, Rosario Alonso-Dominguez, Hsiao-Yean Chiu, Pei-Shan Tsai, Hsin-Chien Lee, Maria I. Rihuete-Galve

**Affiliations:** ^1^Facultad de Enfermería y Fisioterapia, Universidad de Salamanca, Unidad de Investigación de Atención Primaria de Salamanca (APISAL), Instituto de Investigación Biomédica de Salamanca (IBSAL), Red de Investigación en Cronicidad, Atención Primaria y Promoción de la Salud (RICAPPS), Salamanca, Spain; ^2^Universidad de Salamanca, Unidad de Investigación de Atención Primaria de Salamanca (APISAL), Instituto de Investigación Biomédica de Salamanca (IBSAL), Salamanca, Spain; ^3^School of Nursing, College of Nursing, Taipei Medical University, Taipei, Taiwan; ^4^College of Humanities and Social Sciences, Graduate Institute of Humanities in Medicine, Taipei Medical University, Taipei, Taiwan; ^5^Facultad de Enfermería y Fisioterapia, Universidad de Salamanca, Instituto de Investigación Biomédica de Salamanca (IBSAL), Hospital Universitario de Salamanca, Salamanca, Spain

**Keywords:** neurofeedback, sleep quality, insomnia, biofeedback, brain waves

## Abstract

**Objective:**

This systematic review and meta-analysis of randomized-clinical trials aims to analyze the effect of interventions incorporating surface neurofeedback techniques on self-perceived sleep quality and insomnia in patients with or without sleep disturbances.

**Methods:**

The review was completed in accordance with the Preferred Reporting Items for Systematic Reviews and Meta-Analysis (PRISMA) statement and was deposited in the Prospero international prospective registry of systematic reviews (CRD42024528401). Seven clinical trials with different main outcomes but with pre-post intervention records of self-perceived sleep quality or insomnia symptoms assessed by questionnaires met our inclusion criteria, including a publication date within the last 10 years. Five trials investigated sleep quality through scores on the Pittsburgh sleep quality Index (PSQI) and three trials signs of insomnia severity assessed with validated scales. The methodological quality of the included studies was assessed using the Cochrane Collaboration’s tool for assessing the risk of bias and showed a high quality of them.

**Results:**

A total of 5 studies that evaluated sleep quality with the PSQI total score were included in the meta-analysis. The results revealed that control conditions succeeded in improving PSQI-assessed sleep quality more than the analyzed Neurofeedback interventions (PSQI total score 0.57; 95% CI 0.13 to 1.01; *p* = 0.01). On the other hand, a total of 3 studies that evaluated insomnia severity with various insomnia scales were included in the meta-analysis The results revealed that neither the NF interventions nor the control conditions show a favorable outcome relative to each other (−0.13; 95% CI −0.44 to 0.18; *p* = 0.41).

**Conclusion:**

The interventions studied mostly apply a neurofeedback training protocol based on maintaining alpha waves in a range between 8 and 12 Hz, with electrode positioning in the frontal area or in the sensorimotor cortex and with a number of neurofeedback sessions ranging from 8 to 20 sessions. The meta-analysis showed that interventions incorporating surface neurofeedback do not produce additional benefits in self-perception of sleep quality or insomnia compared to a wide variety of control conditions including cognitive behavioral treatment or other biofeedback modalities.

**Systematic review registration:**

PROSPERO – International prospective register of systematic reviews – CRD42024528401 https://www.crd.york.ac.uk/prospero/display_record.php?RecordID=528401.

## 1 Introduction

Sleep quality is a term used to define an individual’s self-satisfaction with all aspects of sleep, which can be categorized into sleep efficiency, sleep latency, sleep duration and wakefulness after sleep onset (Nelson et al., 2022). Possible determinants of sleep quality include lifestyle factors (physical activity, diet, toxic habits or time spent in front of screens), psychological factors (anxiety, depression or stress), presence of certain morbidities or medication, environmental and sociodemographic factors (family income, employment status, living conditions of the usual dwelling, ambient temperature) and other biological factors (e.g., the level of melatonin, cortisol or vitamin D) (Jiménez-Vaquero et al., 2023).

Good sleep quality has positive effects such as feeling rested, maintaining a good cognitive state and normal personal relationships. The consequences of poor sleep quality include fatigue, irritability, daytime dysfunction, slowed responses and increased caffeine/alcohol consumption, as well as possible impacts on aspects related to personal motivation and quality of life (Nelson et al., 2022). In addition, the sleep duration has shown an increased risk of cardiovascular disease and higher all-cause mortality rates, with a U-shaped association in those individuals with a daily total sleep time less than 6 h and more than 8 h (Wang et al., 2019). Meanwhile, insomnia, characterized by difficulty with sleep onset, maintenance, and subsequent daytime symptoms, is increasingly prevalent and increases the risk of other medical comorbidities (Paul and Salas, 2024).

The study of sleep quality includes a wide variety of methods, among which polysomnography stands out for its accuracy. However, polysomnography is a complex and costly test in economic terms, which makes it difficult to include in research studies. The proliferation in recent years of different actigraphy devices, which have a sensitivity of over 90%, means that these devices are used in longitudinal and epidemiological studies (Ibáñez et al., 2019; Zinkhan and Kantelhardt, 2016). However, preliminary sleep assessment is usually completed with sleep questionnaires or sleep scales. Sleep questionnaires are a very cheap and quick test and, moreover, they summarize quantitatively the patient’s (subjective) perception of his or her own sleep quality. However, their subjectivity does not necessarily make the questionnaires inaccurate, as several validation studies have shown. The accuracy of sleep questionnaires has been extensively studied (Silva et al., 2011). All of these studies used polysomnography as the gold standard. The reported sensitivity was in the range 73–98%, while the reported specificity was in the range 50–96%. In addition, for some variables such as total sleep duration, the difference between the results of a questionnaire and the actigraphy measurement seems to be minimal (Kanda et al., 2023).

Neurofeedback (NF) is a form of biofeedback training that uses the recording of brain activity through imaging techniques to achieve, through a process of feedback, control and regulation of brain activity patterns. Based on the principles of operant conditioning, patients learn gradually through positive reinforcement provided by feedback (Marzbani et al., 2016). There are several types of NF, being the most commonly used frequency/power NF, also known as “surface neurofeedback.” This technique consists of placing surface electrodes (usually 2–4 in number) on the individual’s head that record the brain’s electrical activity. This activity is analyzed by a computer program that converts the EEG waves into visual, auditory or tactile signals that it sends to the patient so that he/she learns to work in a specific wave range, thus achieving the regulation of brain activity. These techniques have been used to change the amplitude or speed of specific brain waves in specific brain locations to treat ADHD, anxiety or insomnia (Banerjee and Argáez, 2017).

The increase in the number of devices capable of performing NF, together with the reduction in the cost of their acquisition, has led to a notable increase in recent years in the number of studies that have addressed the effect of these techniques on sleep quality. Lambert-Beaudet et al. (2021) published a review analyzing the effect of NF techniques in the treatment of insomnia. They concluded that, although the studies concerning NF as a treatment for insomnia are encouraging, many methodological barriers remained to be resolved in order to prove its efficacy unequivocally. However, this was not a systematic review, it was focused on NF in the treatment of insomnia and did not analyze the quality of the studies.

The objective of this systematic review was to analyze the effect of interventions incorporating surface NF techniques on self-perceived sleep quality and severity of insomnia by analyzing the results and quality of recent randomized clinical trials (RCTs).

## 2 Materials and methods

This systematic review and meta-analysis of RCTs was carried out following the protocol described in the Preferred Reporting Items for Systematic Reviews and Meta-Analysis: (PRISMA) statement (Moher et al., 2009). The protocol was registered with PROSPERO, the International Prospective Register of Systematic Reviews (Registration no. CRD42024528401). The review and meta-analysis were conducted between March and May 2024.

### 2.1 Literature sources

A structured search of electronic databases (Pubmed, Scopus, Cochrane Library, PsycInfo) was performed. Studies were restricted to the last 10 years (from March 2014 to March 2024). Two reviewers (MFC and JIR) performed a search to identify RCTs that studied the effect of NF on sleep quality and insomnia as primary or secondary outcome. The search strategy followed the PICO framework, using key words, free text, and MeSH terms as appropriate and combining Boolean operators of (AND/OR/NOT/quotation marks/brackets). More details of the terms used in the search can be found in Supplementary Table S1. Filters were applied to limit the search to RCTs and the English language.

### 2.2 Eligibility criteria

This study was guided by the participants, interventions, comparisons, outcomes and study design (PICOS) framework.

#### 2.2.1 Study design

The eligible study design was randomized controlled trials or crossover trials with random assignment to the established sequence to the different arms of the study. Observational studies (case–control, prospective cohort, cross-sectional studies, case reports and case series) and non-randomized clinical trials or trials without a control group were excluded.

#### 2.2.2 Population

The population of interest was adults or young people over 18 years of age, with no distinction of sex. Studies of patients with insomnia or sleep disturbances and studies that included individuals without the presence of these processes were included.

#### 2.2.3 Intervention (exposure)

The exposures of interest included any interventions that included surface NF regardless of the protocol used, the number of sessions or the site of electrode placement. Interventions where other NF models such as Z-score neurofeedback or functional magnetic resonance imaging (fMRI) neurofeedback were performed were excluded.

#### 2.2.4 Comparison

All studies with a control group were selected, regardless of the type of comparison, not only placebo control.

#### 2.2.5 Outcomes

Eligible outcomes were evaluated for the presence of global sleep quality score data or insomnia severity score data assessed through questionnaires. We excluded studies that did not provide global data in both pre- and post-intervention assessments. In addition, other sleep quality-related indicators such as total sleep time, sleep latency, sleep efficacy, wakes after sleep onset and sleep satisfaction were analyzed, whenever present.

### 2.3 Study selection

**Figure 1:**
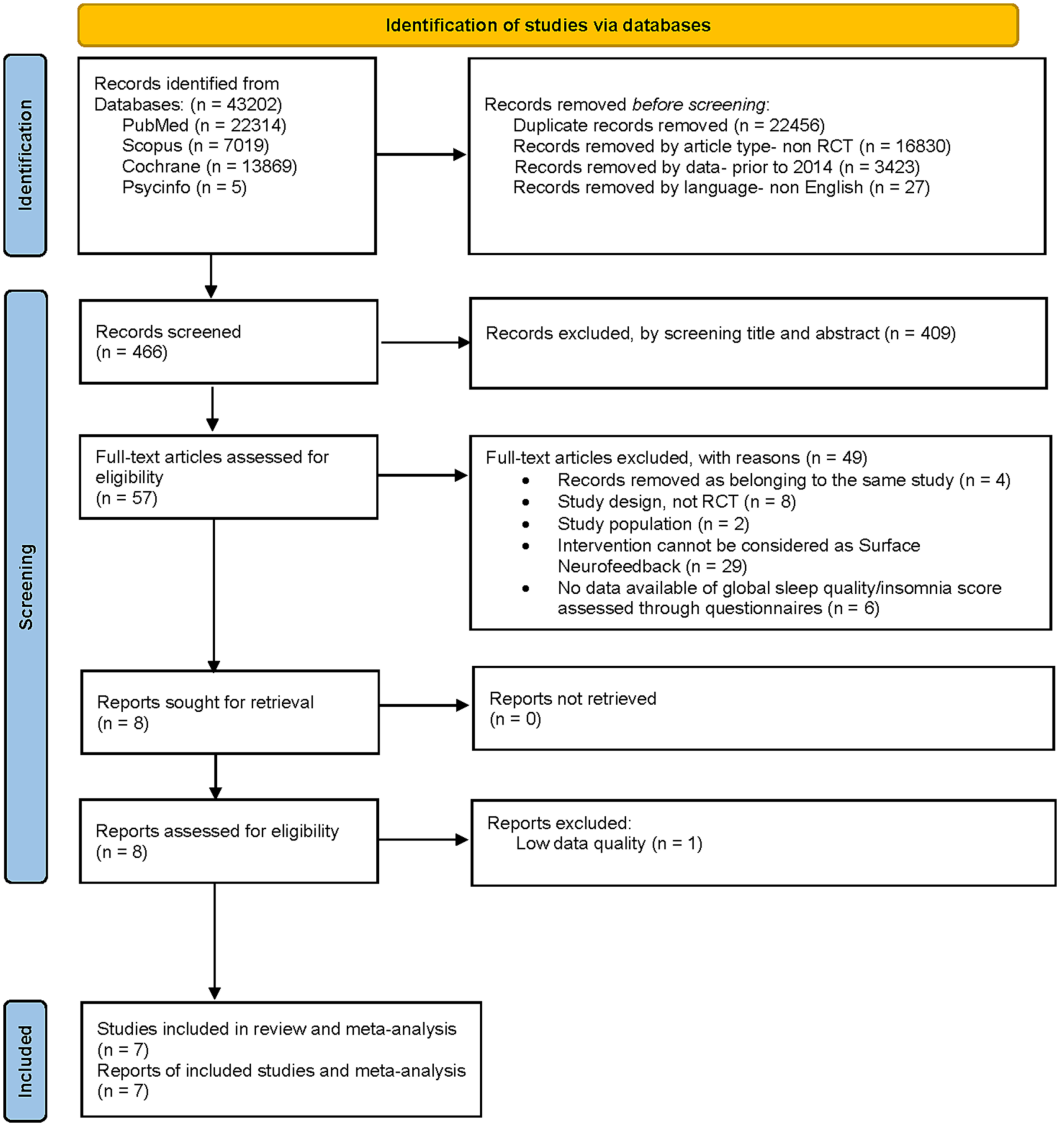
PRISMA 2020 flow diagram for the systematic review.

The titles and abstracts of the retrieved literature were first downloaded and imported into Endnote X9 (Clarivate; a reference management program) to eliminate duplicates. After removing duplicates and applying article type, data, and language filters, two reviewers (MFC and JIR) independently evaluated the article titles and abstracts. At least one reviewer selected studies for full-text review. The full texts of the included studies were independently reviewed by two reviewers (MFC and JIR). During this phase, records were excluded in application of the selection criteria described in the PICO framework. Disagreements were resolved by discussion and by decision of a third reviewer (IGY) when necessary (Figure 1).

### 2.4 Data extraction

Three reviewers (MFC, JIR, and IGY) independently extracted data into a predesigned table in Microsoft Excel. If a study had multiple publications, the most recent one with completed data was selected. Study characteristics such as reference (first author), year, country, study population (health conditions, age, sex), type of RCT, sample size, main and secondary outcomes, and duration were extracted and recorded in Table 1.

**Table 1:** Study characteristics.

References	Year, country	Population	Type of RCT	Sample size	Principal outcome	Secondary outcomes	Duration
Hsueh et al.	2016, Taiwan	Healthy young adults 19–29 years 60% females	Parallel (2 arms)	N = 50 IG = 25 CG = 25	Memory tasks	Cognitive function Depression Anxiety Sleep quality	4 weeks
Kwan et al.	2022, Republic of Korea	Patients with insomnia Average age: 25 years 65% females	Parallel (2 arms)	N = 17 IG = 9 CG = 8	Sleep quality Insomnia	Anxiety	Not specified
Leem et al.	2021, Republic of Korea	Patients with Post-Traumatic Stress Disorder 20–55 years 90% females	Parallel (2 arms)	N = 22 IG = 11 CG = 11	Post-traumatic stress Disorder symptom	Anxiety Depression Insomnia Quality of life Cost outcomes	12 weeks
Li et al.	2024, China	Highly trained athletes with sleep disturbances Average age: 21 years 100% males	Crossover (2 conditions)	N = 14 IG = 14 CG = 14	Sleep quality	Mood states Reaction time	8 weeks
Min et al.	2023, Republic of Korea	Patients with Perceived Stress 19–65 years 90% females	Parallel (3 arms)	N = 94 IG = 30 CG^1^ = 33 CG^2^ = 31	Perceived stress	Mindfulness Insomnia Depression	8 weeks
Schabus et al.	2017, Austria	Patients with insomnia (n = 16) or with insomnia misperception (n = 9) Average age: 39 years 63% females	Crossover (2 conditions)	N = 25 IG = 25 CG = 25	Sleep quality	Sleep dependent Memory consolidation Anxiety Depression	14 weeks
Wu et al.	2021, Taiwan	Patients with fibromyalgia 21–82 years 89% females	Parallel (2 arms)	N = 80 IG = 60 CG = 20	Pain and fibromyalgia impact	Sleep quality Cognitive function	8 weeks

IG, Intervention/neurofeedback group; CG, Control group.

### 2.5 Data analysis

Review Manager v.7.7.2 (The Cochrane Collaboration) was used to perform the statistical analyses. For studies with multiple measurements, only data from baseline and the immediate post-intervention time were extracted for analysis. If the necessary data were not reported, the first/corresponding authors of the relevant publication were contacted. For continuous outcomes, mean values and their SD were used in meta-analyses. Mean differences and 95% CI were used to assess the effect of NF interventions on the Pittsburgh Sleep Quality index total score while standardized mean differences (SMD) and 95% CI were calculated to analyze the effect of NF interventions on the insomnia scales total score, because this variable was collected through different tools in the studies analyzed. Finally, forest plots were generated.

Heterogeneity testing and the meta-analysis were conducted. A 2-sided *p* value <0.05 was considered statistically significant. Heterogeneity was evaluated using the χ^2^ test (with *p* < 0.10 indicating heterogeneity) and I2 test (with I2 > 50% indicating moderate heterogeneity and I2 > 75% indicating high heterogeneity). If I2 ≤ 50% and *p* > 0.10, a fixed-effect model was adopted for data merging and analysis; otherwise, a random-effects model was used.

### 2.6 Quality assessment (risk of bias)

Two evaluators independently assessed the methodological quality of the included studies using the Cochrane Collaboration’s tool for assessing the risk of bias (Higgins et al., 2023). For all RCTs, the following aspects were assessed: (Nelson et al., 2022) Randomization process (Jiménez-Vaquero et al., 2023) Deviations from the intended interventions (Wang et al., 2019) Missing outcome data (Paul and Salas, 2024) Measurement of the outcome (Ibáñez et al., 2019) Selection of the reported result. In addition, for crossover trials only, we assessed one more aspect: Bias arising from period and carryover effects. Each study was categorized as “low risk,” “uncertain risk,” or “high risk,” with disagreements resolved through consultation or discussion with a third researcher.

## 3 Results

### 3.1 Search results and selection

The preliminary search in all databases analyzed yielded 43,202 records. After removing 22,456 duplicated results and filtering by article-type, data and language, leaving 466 records for the screening. After the screening by title and abstract, 57 full-text articles were assessed for eligibility. A total of 49 of them were excluded by applying selection criteria: 4 records removed as belonging to the same study, 8 because of their study design, not RCT, 2 because of the characteristics of their study population, 29 because their intervention could not be considered as a “Surface neurofeedback” technique and 6 for not having available data of global sleep quality/insomnia score assessed through questionnaires. Finally, 8 records were assessed for eligibility although one was eliminated due to low quality data. A total of 7 records from 7 studies were included in the systematic review, 5 of which were included in the meta-analysis of the effect of NF techniques on the global sleep quality score assessed with the PSQI, and 3 of which were included in the meta-analysis of the effect of NF techniques on the total score on insomnia severity scales. The screening process is detailed in Figure 1.

### 3.2 Study characteristics

Table 1 summarizes the main characteristics of the included studies. These studies were all RCTs, 5 of them of parallel type (4 with 2 arms and one with 3 arms) and 2 of them of crossover type with 2 conditions and with randomization of the sequence. The studies are conducted in: The Republic of Korea (Wang et al., 2019), Taiwan (Jiménez-Vaquero et al., 2023), China (Nelson et al., 2022) and Austria (Nelson et al., 2022) between the years 2016, the oldest and 2024 the most recent. Three of these studies include population with insomnia or sleep disturbances. The rest of the studies included healthy population, with perceived stress or post-traumatic stress and people with fibromyalgia. The population range was variable but always older than 18 years, with a predominance of young or young-adult population, all of them having a percentage of women above 60%. Only 3 of the included studies had as principal outcome (Li et al., 2024; Kwan et al., 2022; Schabus et al., 2017) the change in sleep quality or insomnia, being present in the other 4 studies as secondary outcome (Hsueh et al., 2016; Leem et al., 2021; Min et al., 2023; Wu et al., 2021).

### 3.3 Study parameters for surface neurofeedback and control conditions

The included studies used a wide variety of devices to perform the NF sessions (Table 2). The placement of electrodes for these sessions, however, was grouped as follows: in three studies electrodes were placed in the sensorimotor cortex and in three other studies they were positioned in the frontal area. Only the work of Leem et al. (2021) used a placement in the parietal area. In relation to the objective of the sessions and the protocol used, the objective of maintaining alpha waves in a range between 8 and 12 Hz predominated, this objective being present in 5 of the 7 studies analyzed. Three of the studies reviewed included SMR wave training (enhance 12–15 Hz brainwaves) in isolation or in combination with the goal of inhibiting beta waves (18–30 Hz). The study by Wu et al. (2021) scheduled different sessions for both objectives (maintain alpha waves and SMR wave training). The number of NF sessions ranged from 8 to 20 sessions, with the exception of the study by Min et al. (2023) in which 56 self-managed sessions (2 per day) were performed. The mean duration of each session was between 20 and 50 min. In relation to the control conditions, the studies showed a great variability. Three studies compared the results of NF techniques against other types of biofeedback (random, HR feedback or placebo feedback) (Li et al., 2024; Schabus et al., 2017; Hsueh et al., 2016). The remaining studies compared against a wide variety of interventions including cognitive behavioral treatment, mindfulness training or usual treatment and lifestyle.

**Table 2:** Study parameters for surface neurofeedback.

	Neurofeedback groups					Control conditions
Reference	Device	Training electrode location	Protocol objective of the sessions	N° sessions	Duration	
Hsueh et al.	Ni USB-6009 + Labview (National Instruments, TX).	C3, Cz, and C4, respectively (C3a, C3p, Cza, Czp, C3a, and C4p).	Maintain alpha wave (8–12 Hz).	12 (3 per week) within 4 weeks	45 min. 2-min EEG baseline recording followed by six training blocks of 6 min with a 1-min break for resting	Amplitude feedback in random frequency bands.
Kwan et al.	Thought Technology’s Procomp 5	F3 and F7	Reduce the absolute power of the beta waves (18–30 Hz). Maintain the sigma wave (12–15 Hz).	10	30 min.	Cognitive-behavioral treatment for insomnia. Six weekly sessions (50 min per session) for sleep education, sleep restriction and sleep hygiene, stimulation control, relaxation therapy, and relapse prevention
Leem et al.	ProComp2, 2-Channel EEG System with version 6.0 Infiniti Software (Thought Technology Ltd., MontrealWest, Quebec, Canada)	Parietal lobe (PZ)	Maintain alpha (8–12 Hz) and theta (4–7 Hz) waves.	16 (2 per week)	50 min. Three sets of training sessions that lasted 10 min, with a 5 min break between each training session and a 10 min finish	Usual treatment and lifestyle.
Li et al.	Sichiray software (Jiangsu Maiding Technology Company) with a ThinkGear Asic module (Neuro Sky Inc., American)	FP1	Maintain alpha waves (8–12.9 Hz).	8 over a period of 15 days	25 min.	Heart Rate Variability Biofeedback.
Min et al.	OMNIFIT Brain; Omni C&S, Inc	FP1 and FP2	Maintain alpha waves (8–12 Hz).	56 self-managed sessions (2 per day) over a period of 4 weeks	20 min.	CG^1^: Mindfulness-based training program (same protocol as IG). CG^2^: Self-care, were given self-learning paper materials on stress management during their first visit, without any additional weekly meetings.
Schabus et al.	Eldith THERA PRAX (neuroConn) system	C3	Maintain the SMR (12–15 Hz)	12 over a period of 2–4 weeks	40 min. Eight 5-min training blocks (with 13–25 trials within each block)	Placebo-feedback (same protocol as IG).
Wu et al.	ProComp Infinity biofeedback device (Though Technology Ltd., Toronto, Canada)	C3, C4, and Cz	*4 sessions* to enhance alpha waves (8–12 Hz) *12 sessions* of SMR wave training (enhance 12–15 Hz brainwaves and simultaneously inhibit theta (4–7 Hz) and beta (18–22 Hz) brainwaves) *4 sessions* to receive either alphawave or SMR wave training according to their preference	20 over a period of 8 weeks	30 min.	Weekly telephone support during the 8-week treatment period. Each telephone call lasted approximately 10 min, with 5 min of questions and answers regarding the educational materials regarding fibromyalgia and a 5-min debriefing.

CG, Control group; EEG, electroencephalogram; Hz, Hertz; IG, Intervention/Neurofeedback group; SMR, sensorimotor rhythm.

The participant interaction during NF sessions in each study to achieve the stated objectives is summarized in Supplementary Table S2.

### 3.4 Risk of bias

**Figure 2:**
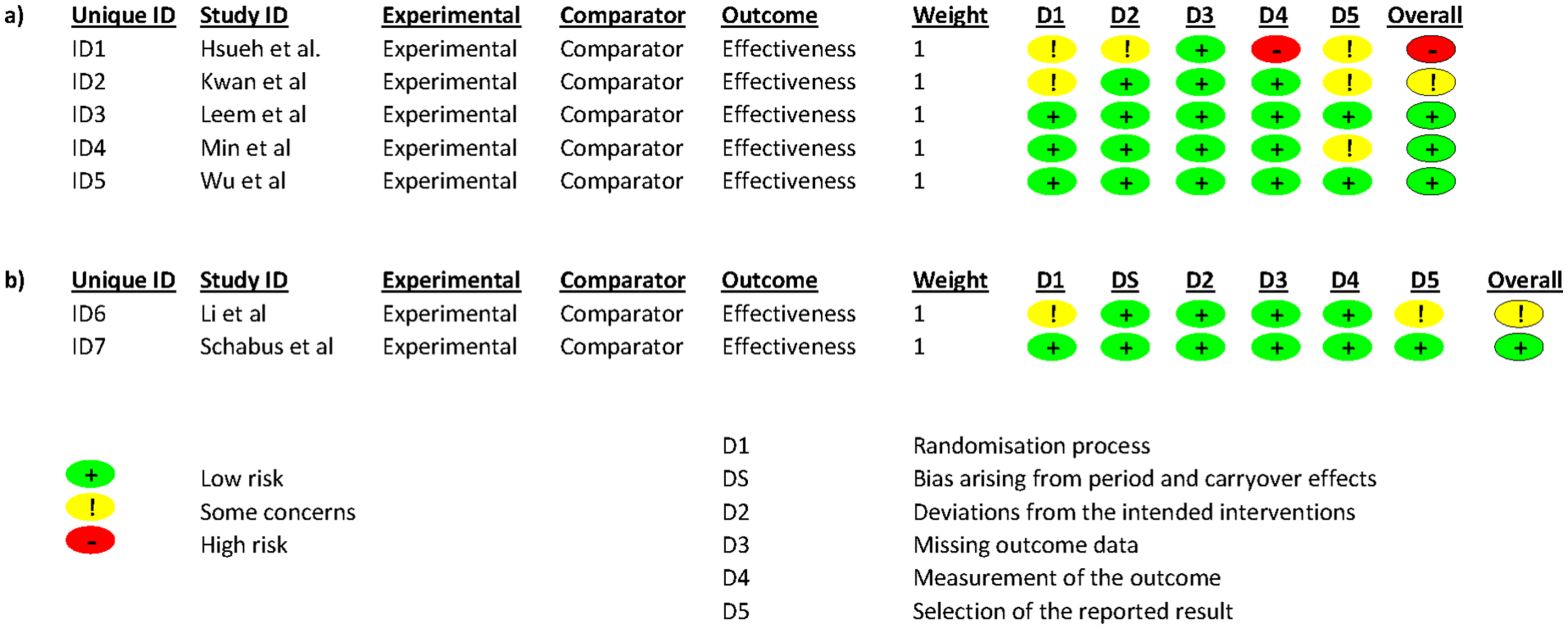
Risk of bias in each study. Red, green, and yellow colors indicate high, low, and unclear risk of bias, respectively.

A quality assessment of the included studies was conducted using the Cochrane Collaboration’s tool (Figure 2). At least 4 of the 7 included studies were considered by the investigators as low risk of bias. The study by Kwan et al. (2022) and that by Li et al. (2024) present some questionable issues. In both studies, it is not known whether the randomization sequence was concealed until the allocation of the interventions and it is not known whether there was an analysis plan established by a research protocol or by the clinical trial registry. The study that raises the most doubts is that of Hsueh et al. (2016). There is little information on the concealment of the randomization sequence until the allocation of the interventions. There is also no information on whether or not the participants and caregivers were aware of the allocation. Nor is it known whether for the sleep variables studied, the statistical analyses were appropriate. It is also not known whether the statisticians in charge of the analysis were blinded or not, and it could be that the fact of knowing the assigned treatment could have influenced the evaluation of the results. Finally, a prior data analysis plan is also unknown in this study.

### 3.5 Meta-analysis

Sleep quality defined with the PSQI total score.

**Figure 3:**
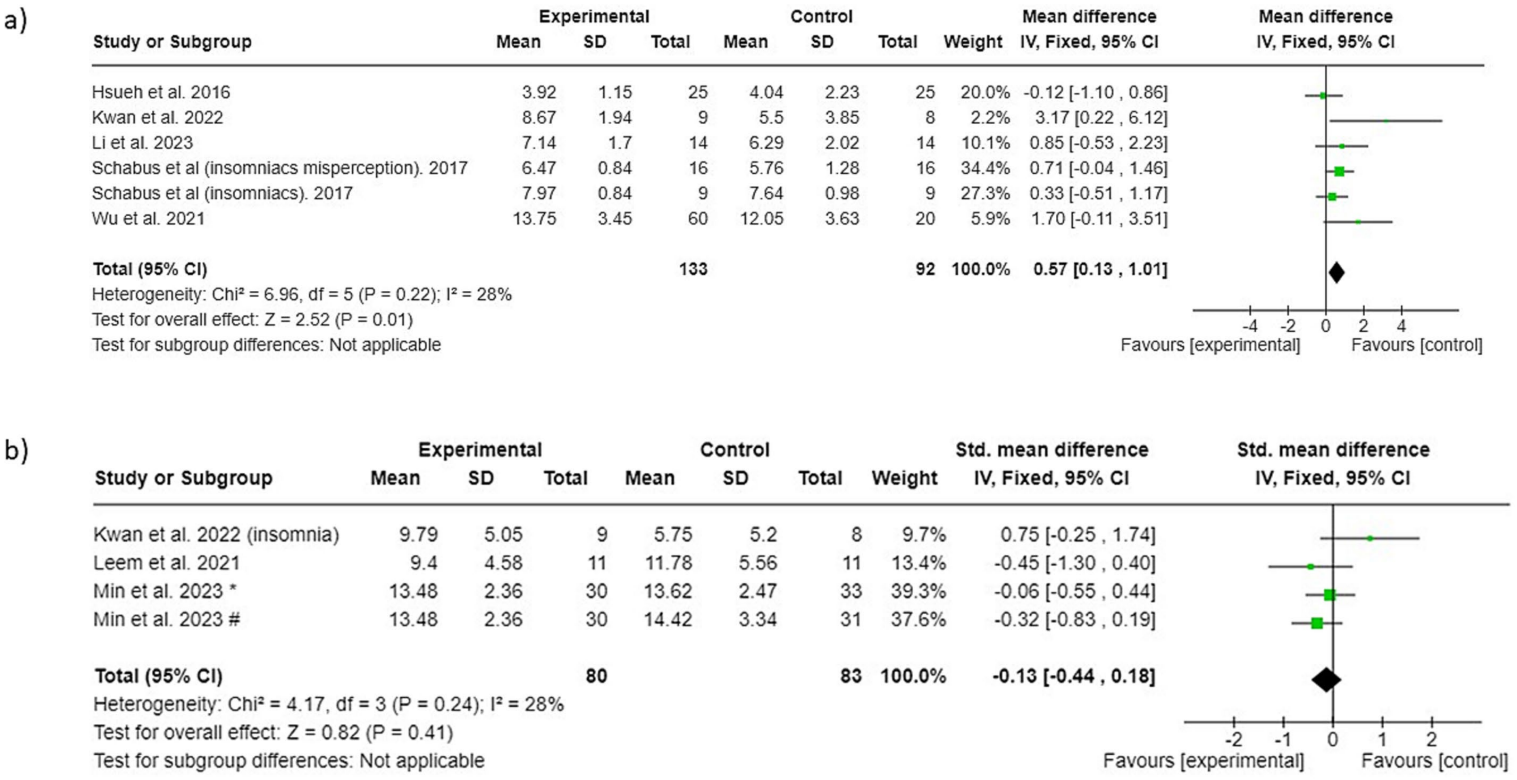
Forest plot of surface neurofeedback interventions versus control conditions: (a) sleep quality (b) insomnia. * NF groups vs. control condition 1, # NF groups vs. control condition 2.

A total of 5 studies that evaluated sleep quality with the PSQI total score were included in the meta-analysis (Li et al., 2024; Kwan et al., 2022; Schabus et al., 2017; Hsueh et al., 2016; Wu et al., 2021). Since no significant heterogeneity was observed among the included studies (I2 = 28%; *p* = 0.22), a fixed-effect model was used for merging the data. The results revealed that control conditions succeeded in improving PSQI-assessed sleep quality more than the analyzed NF interventions (PSQI total score 0.57; 95% CI 0.13 to 1.01; *p* = 0.01), as shown in Figure 3a.

The results of the mean scores on the PSQI pre and post-immediate time can be seen in Supplementary Table S3.

Insomnia severity defined with the insomnia scales total score.

A total of 3 studies that evaluated insomnia severity with various insomnia scales were included in the meta-analysis (Kwan et al., 2022; Leem et al., 2021; Min et al., 2023). Since no significant heterogeneity was observed among the included studies (I2 = 28%; *p* = 0.24), a fixed-effect model was used for merging the data. The results revealed that neither the NF interventions nor the control conditions show a favorable outcome relative to each other (−0.13; 95% CI −0.44 to 0.18; *p* = 0.41), as shown in Figure 3b.

The results of the mean scores on the insomnia scales pre and post-immediate time can be seen in Supplementary Table S4.

### 3.6 Other sleep quality indicators

Table 3 shows the results and intra- and intergroup differences in other sleep quality indicators. Only the study by Li et al. (2024) analysed differences in the scoring of the dimensions of which the PSQI is composed, showing an intra-group difference within the NF intervention groups in the dimensions of sleep duration, with better scores in the post-intervention assessment.

**Table 3:** Other sleep quality indicators.

References						
	PSQI derived sleep indicators					
	Subjective sleep quality	Sleep latency	Sleep duration	Sleep efficiency	Sleep disturbances	Daytime dysfunction
Li et al.	IG (*p* = 0.008) CG (*p* > 0.05) Intergroup difference *p* > 0.05	IG (*p* > 0.05) CG (*p* > 0.05) Intergroup difference *p* > 0.05	IG (*p* = 0.034) CG (*p* = 0.005) Intergroup difference *p* < 0.05	IG (*p* > 0.05) CG (*p* > 0.05) Intergroup difference *p* > 0.05	IG (*p* > 0.05) CG (*p* > 0.05) Intergroup difference *p* > 0.05	IG (*p* > 0.05) CG (*p* > 0.05) Intergroup difference *p* > 0.05
	Sleep-related indicators					
	Total sleep time	Time in bed	Sleep efficacy	Sleep latency	Wake after sleep onset	Sleep satisfaction
Kwan et al.	IG (*p* = 0.008) Pre 387.00(77.97) Post 459.28(95.48) CG (*p* = 0.092) Pre 368.91(86.32) Post 419.29(47.58) Intergroup difference *p* > 0.05	IG (*p* = 0.110) Pre 456.79(81.38) Post 493.49(116.12) CG (*p* = 0.035) Pre 503.69(53.18) Post 453.67(25.13) Intergroup difference *p* > 0.05	IG (*p* = 0.011) Pre 84.07(6.55) Post 92.46(3.57) CG (*p* = 0.012) Pre 76.9(12.63) Post 92.42(9.29) Intergroup difference *p* > 0.05	IG (*p* = 0.038) Pre 43.22(20.58) Post 25.89(16.40) CG (*p* = 0.207) Pre 56.04(34.41) Post 31.13(21.74) Intergroup difference *p* > 0.05	IG (*p* = 0.398) Pre 12.78(15.36) Post 7.22(7.63) CG (*p* = 0.035) Pre 17.04(17.46) Post 3.25(6.82) Intergroup difference *p* > 0.05	IG (*p* = 0.011) Pre 4.92(1.27) Post 6.50(1.04) CG (*p* = 0.012) Pre 5.00(1.58) Post 6.46(0.46) Intergroup difference *p* > 0.05
Wu et al.				IG (*p* < 0.05) Pre 51.42(58.78) Post 31.03(29.35) CG (*p* > 0.05) Pre 27.50(18.53) Post 31.68(32.60) Intergroup difference *p* = 0.006		

IG, Intervention/neurofeedback group; CG, Control group.

Kwan et al. (2022) found no intergroup differences in the variables analysed, although intragroup differences within the NF groups were observed in the variables of total sleep time, sleep efficiency, sleep latency and sleep satisfaction. In contrast, the work of Wu et al. (2021) shows better sleep latency (time elapsed between turning off the light and the onset of the first sleep phase) at the post-intervention visit in the NF group compared to the control conditions.

## 4 Discussion

### 4.1 Principal findings

To our knowledge, this is the first study to conduct a systematic review and meta-analysis addressing the effect of surface NF techniques on self-perceived sleep quality and severity of insomnia by analyzing the results and quality of recent RCTs. The number of clinical trials selected and analysed is still limited, although it has grown in recent years. However, the selected studies are generally of good scientific quality, although small in relation to their sample size. The main results of the systematic review and meta-analysis do not show beneficial results of these surface NF techniques for the improvement of self-perceived sleep quality, with the groups used as control condition showing a slightly favorable relationship, as a consequence of the diversity and intensity of the interventions used as control. In relation to the self-perception of the severity of the signs of insomnia, neither the surface NF groups nor the groups used as control condition show a favorable relationship.

### 4.2 Characteristics of the analyzed studies

As shown by the heterogeneity results of the meta-analyses, the studies analyzed are moderately homogeneous and of contrasted quality. However, there are some issues that should be taken into consideration when making an adequate interpretation of the results. The first is that the number of studies analyzed (*n* = 7) and the total number of participants (*n* = 302) are still limited. In addition, most of the studies were conducted in 3 countries (Korea, Taiwan and China) with an Eastern population and only one study worked with a Western population. On the other hand, the health conditions of the populations included in the studies include both people with sleep disorders and people without these problems but with other processes that can affect the perception of sleep quality, such as perceived stress or fibromyalgia (Huang et al., 2024; Tafoya et al., 2023; Osorio et al., 2006; Wu et al., 2017). Finally, there is a clear deviation in the biological sex of the participants included in the different studies, with the majority being women.

### 4.3 Study parameters for surface neurofeedback and control conditions

Most of the studies analyzed (5 out of 7) use a NF training protocol based on maintaining alpha waves in a range between 8 and 12 Hz. The main characteristic of this brain rhythm is its association with the visual system, recorded mainly in the occipital area, which is clearly increased when we close our eyes (Morrone and Minini, 2023). Alpha brain waves are usually associated with relaxed and pleasant moods and are therefore used in the process of relaxation (muscle relaxation), which eventually leads to sleep. Alpha training is often used for the treatment of various conditions, such as pain relief, stress and anxiety reduction, memory improvement, mental performance enhancement and treatment of brain injuries (Lentz et al., 1999; Vernon, 2005).

There is no uniformity in the placement of electrodes for these sessions depending on the objectives of each particular study or the particularities of each device used. The placement of the electrodes for training is of vital importance to achieve appropriate results. It has been described, for example, that training along the right sensorimotor hemisphere of the right hemisphere (C4) can invoke feelings, emotions or calmness and increase concentration. Training on the opposite side (C3) could lead to undesired results such as a depletion of mental energy (Marzbani et al., 2016; Carrobles, 2016; Ribeiro et al., 2023).

Finally, none of the studies analyzed explored the minimum number of sessions necessary to achieve certain beneficial results in relation to sleep quality or the improvement of signs of insomnia. It does seem that all the interventions coincide in proposing an intensive protocol with at least 2 or 3 sessions per week for periods of at least 4 weeks. Interestingly, in this regard, the work of Min et al. (2023) planned a total of 56 self-managed sessions (2 per day) over a period of 4 weeks. The approach of self-managed sessions may increase adherence to the intervention, making it possible to implement this type of intervention in a wider population.

### 4.4 Sleep quality and insomnia

In the groups studied in this review that used NF, all of them obtained a discrete improvement in their final PSQI score. But when this change was compared against control conditions the results did not offer an additional benefit. On the contrary, the results of the meta-analysis conclude that, in the case of self-perceived sleep quality through the PSQI, the groups used as control conditions obtain more favorable results than the NF groups. This finding requires a deep reflection on the possible reasons underlying this relationship. In this meta-analysis, a total of 5 studies were included that compared each NF intervention against different control conditions in each study. These control conditions ranged from placebo feedback or heart rate feedback to intensive cognitive behavioral treatment interventions or intensive telephone support. Intensive interventions with cognitive behavioral treatment have shown very beneficial results on sleep quality when tested independently (De Niet et al., 2009; Barrios Araya et al., 2023; Schramm et al., 2016). The same has been reported for other interventions incorporating biofeedback, e.g., heart rate biofeedback (Li et al., 2022). Another aspect to consider is that the quality of sleep assessed with the PSQI total score reflects multiple aspects of sleep quality. And these aspects can have a very variable result if we analyze them independently. For example, the only study that analyzes the response of the interventions on the different dimensions of the PSQI is the work of Li et al. (2024). The intervention analyzed in this work obtains favorable results in subjective sleep quality and sleep duration. Therefore, future work should analyze which particular aspects of sleep quality may benefit from these NF techniques.

The results extracted from this review show that, although the use of NF seems encouraging for the treatment of insomnia, there are few studies in this field of research. In the 3 studies analyzed, the groups that used NF techniques achieved more beneficial scores on self-perceived severity of insomnia after the intervention, but the changes in these scores were not significant with respect to the groups used as control conditions. In 2021, this relationship has already been analyzed in a review by Lambert-Beaudet et al. (2021) which concludes exactly the same as in this work. Lambert-Beaudet et al. (2021), however, did not perform a meta-analysis of RCTs nor did they perform a systematic review, but among the possible reasons described for not finding satisfactory results are a are many of the ones presented here: (1) Lack of consensus in the protocols used: In two of the three studies an alpha wave maintenance training protocol was used and in another study a maintain of the sigma waves protocol (12–15 Hz). In addition, electrode positioning was also variable between frontal and parietal electrode positioning. There was more agreement on the minimum time of each session, at least 10 min, but not on the number of sessions, with two papers with less than 20 sessions, despite some researchers suggesting that up to 40 sessions of NF are necessary to effectively change behavior and symptoms (Thibault et al., 2017) (2) Small sample sizes: With the exception of the study by Min et al. (2023) (*n* = 94), the rest of the studies had a very small sample (17 and 22 participants) (3) Insufficient placebo control: The control conditions, as with the studies included in the meta-analysis of sleep quality, were very variable. Only the work of Leem et al. (2021) compared their intervention against the usual treatment and it is in this work where the most favorable results for the NF groups are found. Only two of the included studies (Schabus et al., 2017; Hsueh et al., 2016) compared NF versus placebo feedback, although, as suggested in the review by Lambert-Beaudet et al. (2021) to develop a truly inactive placebo treatment will require improvements to those currently in use in research. (4) Possible bias: In contrast to the review by Lambert-Beaudet et al. (2021), in our meta-analysis the studies included, due to the selection criteria used, allow us to highlight the high quality of the RCTs.

### 4.5 Strengths and limitations

This systematic review and meta-analysis of RCTs was carried out following the protocol described in the Preferred Reporting Items for Systematic Reviews and Meta-Analysis: (PRISMA) statement. The search for studies was carried out in several large databases with broad search terms related to possible papers dealing with the topic, although there may have been some papers that could not be found and analyzed. The review only included Surface neurofeedback-type interventions, so there may have been some mixed interventions or interventions not sufficiently described that were not included. However, this is the most common type of NF and with the widest range of devices available to carry it out. The inclusion of studies with data on sleep quality or self-perceived insomnia through validated questionnaires has allowed us to perform a meta-analysis and has made it possible to include studies with a moderate level of heterogeneity. However, these results are self-perceived by the participants, which entails possible biases in the response of the participants in each study. In six of the seven clinical trials included in this work, in addition to the self-reported sleep assessment questionnaires, physiological sleep assessments have been used, which might provide additional important information regarding the effectiveness of NF for sleep quality.

Finally, moderators could not be explored due to small sample sizes of the included RCTs, and inability to detect moderation effects. Also, the differences in electrode placement, NF protocols, and control groups across studies limit the ability to draw solid conclusions.

### 4.6 Implications for practice and future research

The results of this review and meta-analysis do not offer sufficient evidence to incorporate surface NF techniques as an alternative in the treatment of insomnia or the improvement of sleep quality due, among other reasons, to the control conditions with which they have been compared. It is therefore necessary to continue with this line of research by carrying out more quality RCTs, with a greater number of participants, with a more uniform protocol and objectives, with a minimum number of sessions or the possibility of using auto-guided sessions, prior training, and comparing against control conditions that include random placebo feedback and focusing on people with previous sleep disorders who may benefit from it, studying the implications on insomnia and all aspects present in the quality of sleep. Furthermore, as noted above, in addition to self-reported sleep assessment questionnaires, physiological sleep assessments have been used, which could provide important additional information on the efficacy of NF for sleep quality.

## 5 Conclusion

This systematic review and meta-analysis demonstrate that interventions incorporating surface neurofeedback do not produce additional benefits in self-perception of sleep quality or insomnia compared to a wide variety of control conditions including cognitive behavioral treatment or other biofeedback modalities. The interventions studied mostly apply a NF training protocol based on maintaining alpha waves in a range between 8 and 12 Hz, with electrode positioning in the frontal area or in the sensorimotor cortex and with a number of NF sessions ranging from 8 to 20 sessions. However, the number of studies found and analyzed is still scarce, and more RCTs with larger samples and with greater uniformity in their protocols and objectives are needed.

## Data Availability

The raw data supporting the conclusions of this article will be made available by the authors, without undue reservation.
