# Supplementary material for: Neurofeedback to enhance sleep quality and insomnia: a systematic review and meta-analysis of randomized clinical trials
Source: Front Neurosci. 2024 Nov 6;18:1450163. doi: 10.3389/fnins.2024.1450163 (PMC11576419; doi:10.3389/fnins.2024.1450163)
Supplement: Supplementary file 1 [file Table_1.DOCX]

| Table S1: Complete database search strategy | | | |
| --- | --- | --- | --- |
| Database | | **Intervention** | **Outcome** |
| Pub Med | Key or MeSH terms | “Biofeedback, psychology” OR “neurofeedback” OR "machine learning" OR "Feedback sensory" OR "Brain waves" | “Sleep initiation and maintenance disorders” OR “sleep quality” OR "wakefulness, classification/physiology" OR "sleep stages, classification/physiology" |
| Scopus | Key or MeSH terms | “Biofeedback” OR “neurofeedback” OR "machine learning" | “Sleep initiation and maintenance disorders” OR “sleep quality” OR "wakefulness" OR "sleep stages " |
| Cochrane Library | Key or MeSH terms | “Biofeedback” OR “neurofeedback” OR "machine learning" | “Sleep initiation and maintenance disorders” OR “sleep quality” OR "wakefulness" OR "sleep stages " |
| Psycinfo | Key or MeSH terms | “Biofeedback” OR “neurofeedback” OR "machine learning" | “Sleep initiation and maintenance disorders” OR “sleep quality” OR "wakefulness" OR "sleep stages " |

| Table S2: Participant interaction during neurofeedback sessions | |
| --- | --- |
| REFERENCE | **Type of interaction** |
| Hsueh et al. | The instantaneous amplitude was given by the means of a horizontal color bar. If the EEG amplitude increased, the bar moved to the right. Otherwise, a decreased EEG amplitude drove the bar left. Participants were instructed to move the bar to a rightmost position and to hold it there as long as possible. |
| Kwan et al. | The bands into narrower segments to fine-tune the neurofeedback target of beta-2 (15–18 Hz), beta-3 (18–25 Hz), and beta-4 (25–30 Hz). The sound of nature was rewarded when the beta-2, 3, and 4 values fell below the threshold while the sigma values rose above the threshold. |
| Leem et al. | Maintain a relaxed state by strengthening the alpha and theta waves and suppressing any beta waves. |
| Li et al. | The primary feedback mechanism of EEG biofeedback was visual, with participants receiving feedback from a floating yoga ball that adjusts its height based on the proportion of alpha waves detected. Specifically, a higher proportion of alpha waves results in an increase in the height of the yoga ball, providing an objective indicator of the participant’s mental state. |
| Min et al. | Positive feedback sound (a 1-second ringing of a bell) when they were in a focused and relaxed state, whereas a negative feedback sound (a 1-second chirping sound of a cricket) would be provided when they were in a distracted and unrelaxed state. |
| Schabus et al. | During the feedback interval, participants had to mentally ‘move’ a compass needle as far to the left as possible reaching the previously fixed threshold represented by a green dot. Subjects were instructed to test appropriate strategies and find their personally most successful approach to mastering the task. They were told that, for example, a combination of relaxation techniques and positive thought might help them exceed the threshold. After the appearance of the reward signal the next trial started with a new 3 s baseline measurement. |
| Wu et al. | During each neurofeedback enhancement trial, the participants were told to relax and concentrate on a computer-animated game, which was designed to discontinue if brain wave amplitudes were outside the desired range. Both visual and audio indicators were displayed, as rewards to condition the participants’ achievements of control over their EEG. |

| Table S3: Pre-post intervention mean scores on the Pittsburgh Sleep Quality Index-PSQI. | |
| --- | --- |
| REFERENCE | PSQI mean scores |
| Hsueh et al. | IG (p>0.05)  Pre 3.96(1.31)  Post 3.92(1.15)  CG (p>0.05)  Pre 4.20(2.18)  Post 4.04(2.23)  Intergroup difference p>0.05 |
| Kwan et al. | IG (p=0.01)  Pre 12.11(3.79)  Post 8.67(1.94)  CG (p=0.01)  Pre 10.00(2.88)  Post 5.50(3.85)  Intergroup difference p=0.02 |
| Li et al. | IG (p=0.003)  Pre 8.50(2.31)  Post 7.14(1.70)  CG (p=0.025)  Pre 7.57(1.91)  Post 6.29(2.02)  Intergroup difference p=0.24 |
| Schabus et al. | Patients with insomnia (n=16)  IG (p=0.478)  Pre 8.63(0.90)  Post 7.97(0.84)  CG (p=0.416)  Pre 8.61(1.12)  Post 7.64(0.98)  Intergroup difference p=0.261  Patients with insomnia misperception (n=9)  IG (p=0.366)  Pre 7.42(0.90)  Post 6.47(0.84)  CG (p=0.383)  Pre 7.13(1.37)  Post 5.76(1.28)  Intergroup difference p=0.193 |
| Wu et al. | IG (p=0.008)  Pre 14.40(3.93)  Post 13.75(3.45)  CG (p=0.265)  Pre 12.20(4.07)  Post 12.05(3.63)  Intergroup difference p=0.406 |

| Table S4: Pre-post intervention mean scores on the Insomnia Severity scales. | | |
| --- | --- | --- |
| REFERENCE | Insomnia severity index-ISI | Athens Insomnia Scale-AIS |
| Kwan et al. | IG (p=0.007)  Pre 18.67(2.73)  Post 9.79(5.05)  CG (p=0.011)  Pre 17.63(2.72)  Post 5.75(5.2)  Intergroup difference p=0.074 |  |
| Leem et al. | IG  Pre 17.30(5.96)  Post 9.40(4.58)  CG  Pre 12.11(6.89)  Post 11.78(5.56)  Intergroup difference p=0.02 |  |
| Min et al. |  | IG  Pre 14.48(3.12)  Post 13.48(2.36)  CG1  Pre 14.50(2.23)  Post 13.62(2.47)  CG2  Pre 14.90(3.35)  Post 14.42(3.34)  Intergroup difference p=0.740 |
